# Effects of Dietary Salt and Boric Acid on Milk Quality in Savak Akkaraman Sheep

**DOI:** 10.3390/ani16020233

**Published:** 2026-01-13

**Authors:** Pelin Beyazgül, Selçukhan Akarsu, Yasin Baykalir, Ülkü Gülcihan Şimşek

**Affiliations:** 1 Department of Food Hygiene and Technology, Faculty of Veterinary Medicine, Firat University, Elazig 23119, Türkiye; 2 Department of Animal Science, Faculty of Veterinary Medicine, Firat University, Elazig 23119, Türkiye; sakarsu@firat.edu.tr (S.A.); gsimsek@firat.edu.tr (Ü.G.Ş.); 3 Department of Biostatistics, Faculty of Veterinary Medicine, Balikesir University, Balikesir 10463, Türkiye; yasin.baykalir@balikesir.edu.tr

**Keywords:** sheep, milk quality, boric acid, feed additives

## Abstract

This study evaluated the effects of varying dietary salt and boric acid addition doses on the milk quality of Savak Akkaraman sheep. A total of 120 animals were as-signed to six treatment groups (*n* = 20): control (C), rock salt (S; 10 g/day), boric acid 20 mg/day (B20), boric acid 40 mg/day (B40), BS20 (20 mg boric acid + 10 g/day rock salt), and BS40 (40 mg boric acid + 10 g/day rock salt). All analyses were performed in duplicate on six samples, taken on days 30 and 35 following the administration of the additives. Physicochemical analyses only showed significant variation in milk pH (*p* = 0.006), while acidity, dry matter, and ash remained unaffected. Strong positive correlations were found among protein, lactose, salt, and density (r > 0.95; *p* < 0.001). Coagulation times differed widely, with the longest being observed in BS20 (995.03 s) and the shortest in BS40 (141.73 s). Among mineral parameters, only selenium levels differed significantly between the treatment groups (*p* < 0.05). No significant differences were found for fat, solids-not-fat, lactose, freezing point, or electrical conductivity. Importantly, boron addition had a significant influence on total casein content (*p* < 0.001). Overall, dietary rock salt and boric acid did not markedly alter the basic milk composition but produced notable physicochemical changes, particularly in coagulation behavior and casein levels, which may influence the technological properties of sheep milk.

## Simple Summary

This research confirms the effects of varying doses of salt and boric acid addition on milk quality in Savak Akkaraman sheep. The physicochemical and elemental changes in the milk of Savak Akkaraman sheep following dietary addition with rock salt and boron were analyzed. Physicochemical analyses revealed differences in clotting time, total casein, and Se content. This method provides important information regarding the composition of this animal’s diet and the nutritional value of its milk.

## 1. Introduction

As the global population is expected to reach 9.8 billion by 2050, the sheep farming sector will play a key role in meeting the demand for animal protein, which is increasing rapidly with population growth, especially in developing countries [1]. To meet this demand and ensure food security in a sustainable manner, the efficiency of production must be increased. One of the most effective strategies is to optimize feed consumption without reducing animal performance, which is possible by enriching the feed. Sheep farming contributes to economic diversification and supports rural development by providing valuable products such as meat, milk, wool, and leather. Additionally, it contributes to environmental sustainability owing to its lower carbon footprint compared with other cattle breeds. With these features, sheep farming will continue to have an important share in both current and future agricultural and food systems [2].

Milk is a natural food secreted by mammals that contains the necessary nutrients for the development of offspring [3]. Drinking milk is mostly consumed in UHT, pasteurized, or enriched forms around the world [4]. The number of small ruminants in Turkey increased by 4.8% in 2024, reaching 54.9 million head, while milk production increased by 4.7%, reaching 22.5 million tons. Sheep and goat milk constitute 1.389.192 tons of this production [5,6]. It is reported that the production of goat and sheep milk constitutes an important part of the national economies of several countries, especially in the Mediterranean and the Middle East, and that production is generally well organized in countries such as France, Spain, Italy, and Greece [7,8].

Sheep milk varies in composition depending on species, breed, nutrition, environmental factors, and lactation stage [9,10]. On average, it contains 7% fat, 6.2% protein, and 4.9% lactose [7]. Sheep milk caseins contain more calcium and are more sensitive to rennet than those in cattle milk [11]. It is also rich in vitamins A and B and minerals [12,13]. In terms of mineral content, goat and sheep milk have higher Fe, Zn, and Cu levels than human milk [14]. The bioavailability of these minerals varies depending on their binding to proteins in milk. Although the number of small ruminants worldwide has generally increased over the long term, this pattern is not consistent across all regions, and certain countries show a decreasing trend. Global sheep and goat numbers have increased by about 1% annually for years. While this trend may be limited or even declining in developed countries, it is more pronounced in some developing nations [15].

Salt (NaCl) plays an important role in fluid–electrolyte balance and blood pressure regulation in the body and is essential for the maintenance of vital physiological functions [16]. Mineral supplementation is essential for the nutrition of sheep and goats. It has been established that providing salt to sheep at regular intervals is necessary for both animal health and product quality, and 15–30% salt is usually added to their diet [17]. Sodium (Na) and chloride (Cl) are essential macrominerals in sheep, playing key roles in cellular osmoregulation, nerve transmission, acid–base balance, and the regulation of water intake. According to the Nutrient Requirements of Small Ruminants [18], a maintenance diet for adult sheep should contain 0.08–0.10% sodium (Na) and 0.10–0.15% chlorine (CI) of dry matter in the total diet. During lactation, these requirements increase substantially due to higher mineral losses through milk and increased water consumption. Reports from the NRC [18] and AFRC [19] indicate that dietary levels of 0.10–0.18% Na and 0.15–0.20% Cl of dry matter in the total diet are considered adequate and safe for lactating ewes. Providing free access to salt (NaCl) is the most practical and effective method for meeting these needs. Symptoms of Na or Cl deficiency may include reduced appetite, decreased milk yield, pica, and impaired growth [20].

Boron (B) is a strategically important element, and Türkiye holds about 72% of the world’s known reserves [21]. It is widely used in various fields, such as industry, agriculture, and medicine. In recent years, it has attracted attention in the food industry due to its potential to prevent microbial spoilage, increase nutritional value, and extend shelf life [22]. Boron is a trace element that must be consumed daily by both animals and humans. The minimum daily requirement has been reported to range between 2.16 and 2.28 mg/day [23]. According to the World Health Organization (WHO) [24], the concentration of boron in drinking water ranges between 0.1 and 0.3 mg/L, while dietary boron intake has been estimated to be approximately 1.2 mg/day. While the WHO has defined a safe daily intake of boron for adults as 1–13 mg/day, the European Food Safety Authority (EFSA), taking toxicokinetic and toxicodynamic studies on boron into account, has set the maximum tolerable daily intake for adults at 10 mg/day for boric acid and boron salts [25]. Based on the adverse effects of excessive boron intake on reproduction and development, the U.S. Food and Nutrition Board has established the tolerable upper intake level (UL) for this trace element to be 20 mg/day [26].

Moreover, boron has emerged as a topic of growing interest in the field of livestock nutrition. In animals, the physiological roles of boron are considered especially important in bone formation, immune responses, and the regulation of mineral metabolism. Some studies on ruminants (e.g., cattle and sheep) have reported that low-level boron addition to feed has a positive effect on the feed conversion ratio and growth performance [27]. In experimental studies on monogastric animals (e.g., chicken and pig), it has been observed that boron supports bone mineralization and contributes to the development of the skeletal system by balancing calcium and magnesium metabolism [28]. Additionally, it has been stated that boron may play a role in reducing oxidative stress by strengthening the antioxidant defense system [29]. These effects demonstrate the potential of boron in protecting animal welfare and increasing productivity, especially in intensive production systems. Collectively, these findings demonstrate that boron has the potential to make a strategic contribution to animal production. However, studies investigating the effects of boron on the yield and quality of milk remain limited.

The main objective of this study was to reveal the changes in milk quality caused by the addition of different amounts of boric acid and salt to the rations of Savak Akkaraman sheep and to scientifically evaluate the possible effects of these additives on milk composition.

## 2. Materials and Methods

### 2.1. Experimental Design and Milk Supply

This study was conducted on a local farm with 510 sheep in the Baskil district of Elazig in Türkiye. Prior to the experiment, all sheep were examined, tagged, and assessed for body condition scores and age. Among these sheep, 120 animals with a similar age, body condition score, and type of birth were selected. The age (2.5–3 years; second lactation), type of birth (single birth), and body condition scores (3–3.5) of the sheep were balanced, and 20 sheep were selected for each group. Furthermore, ear tags with distinct colors were used to identify each group. The treatment groups were housed in six separate pens. Each sheep was allocated approximately 1.60 m^2^ of space in the enclosure, with a feeder length of 50 cm per animal. The sheep were fed with barley (500–550 g/sheep) and wheat straw (600–700 g/sheep) during the experiment. All animals were fed a barley-straw mixture using a group-feeding system. Drinking water was offered ad libitum. The levels of salt and boron in the drinking water, straw, and barley were not considered across the groups. The treatment groups were formed as follows: control group (C): 40 mL of drinking water; rock salt group (S): 10 g/day of rock salt; boric acid group-20 (B20): 20 mg of boric acid/day; boric acid group-40 (B40): 40 mg of boric acid/day; boric acid + rock salt group-20 (BS20): 20 mg of boric acid + 10 g/day of rock salt; and boric acid + rock salt group-40 (BS40): 40 mg of boric acid + 10 g/day of rock salt. The additives were prepared daily as stock solutions (20 sheep × additives × 40 cc drinking water) and orally administered at 40 cc to each sheep at 10:00 a.m. using a 50 cc syringe. To ensure that each sheep received the prescribed dose, the additives were administered individually. The additives were presented to the sheep starting in early August, and milk samples were collected in duplicate from different randomly selected sets of six sheep on August 30 (day 30) and September 4 (day 35). To account for the risk of reduced milk production, a few extra sheep were initially included in the trial. To determine the boron dose that was to be administered to the animals during the treatments, recommendations from the literature [23,24,25,30,31] were used. Boron levels were calculated with the molecular weight of boric acid (H_3_BO_3_ = 61.83 g/mol) in mind. The elemental boron level corresponds to approximately 7 mg/day in the B40 group and 3.5 mg/day in the B20 group. Salt levels were determined in accordance with manufacturer recommendations and the literature [17].

All the milk from each animal in the groups was milked by hand, and after thorough mixing to ensure homogeneity, milk samples were collected in 50 mL tubes. The samples were then immediately transported to the laboratory under cold-chain conditions (+4 °C). A total of six samples per group were processed, with each sample being run in two replicates.

### 2.2. pH, Acidity, Dry Matter, Ash, and Fat Content Measurements

The pH values of the samples were measured with a pH meter (Thermo Scientific Orion Star A 111, Thermo Fisher Scientific, Waltham, MA, USA), calibrated using pH 4 and 7 buffer solutions. The total titratable acidity (% lactic acid) of the samples was determined by the titration method. Dry matter and ash contents were determined by the gravimetric method [32]. The fat content in the raw milk samples was determined according to the Gerber and TS ISO 19662 [33] methods.

### 2.3. Determination of Coagulation Time

Rennet strength was evaluated based on the amount of milk that was ready for fermentation. Yeast strength refers to the quantity of curd that is formed by one unit of rennet within 40 min from freshly milked milk at a fermentation temperature of 35 °C [34]. The clotting time is described as the time needed for visible clots and was determined using the method described by Berridge [35]. In this study, to determine the yeast strength, a 50 mL sample of fresh milk was taken, and 1 mL of rennet (Grade 2, strength: 1/8500) (Yayla, Maysa, Istanbul, Türkiye), diluted 1:10 with water, was added. The milk was gently stirred, and a stopwatch was started to record the time until the first visible curds appeared. Once the coagulation time was recorded, the rennet strength was calculated using the following equation [36]:

Yeast strength = (2400 × S)/(Z × M)

where S: milk volume (mL); Z: coagulation time (s); and M: amount of yeast (mL).

### 2.4. Determination of Lactoscan Analyses

The fat, protein, lactose, solids-not-fat (SNF), density, water content, electrical conductivity, and salt content of the raw milk samples were analyzed using a Lactoscan milk analyzer (Milkotester Master Classic LM2-P1, Milkotester LTD, Belovo, Bulgaria) [37].

### 2.5. Determination of Mineral Substance Analyses

The concentrations of boron (B), calcium (Ca), copper (Cu), iron (Fe), magnesium (Mg), manganese (Mn), molybdenum (Mo), selenium (Se), and zinc (Zn) were analyzed at the Central Laboratory Application and Research Center of Bingöl University. Milk samples were collected from each experimental group and stored at −20 °C until analysis. Prior to mineral determination, samples were thawed at room temperature and homogenized. Mineral analysis was performed after wet digestion using a mixture of nitric acid and hydrogen peroxide. The concentrations of boron and other minerals were quantified using inductively coupled plasma optical emission spectrometry (ICP-OES). Calibration was carried out using certified standard solutions, and quality control samples were analyzed periodically to ensure analytical accuracy. All measurements were performed in triplicate, and results were expressed as mg/kg of milk [38].

### 2.6. Determination of Casein Fractions by SDS-PAGE Method

The milk samples were first homogenized by adding 25 mM of Tris buffer to them (pH 7.4). The total protein content of the milk was determined using a NanoDrop spectrophotometer (Thermo, NanoDrop 2000c, Thermo Fisher Scientific, Waltham, MA, USA), and equal amounts of protein were subjected to sodium dodecyl sulfate polyacrylamide gel electrophoresis (SDS-PAGE). For gel preparation, a stacking gel and a separating gel were cast according to the standard Laemmli [39] method. Milk protein samples were mixed with loading buffer, boiled for 5 min at 95 °C, and then loaded onto the gel. Electrophoresis was performed at a constant voltage until the dye front reached the bottom of the gel. After separation, gels were stained with Coomassie Brilliant Blue R-250, destained, and imaged. Band intensities corresponding to total casein were quantified using ImageJ software ver.1.51, and the results were expressed as optical density (%OD).

### 2.7. Statistical Analyses

Prior to statistical analysis, data were tested for normality and homogeneity of variances. When the assumptions of normal distribution and equal variances were met, one-way ANOVA was used to evaluate the effects of boric acid, salt, and their combination on the milk’s composition and chemical properties. In cases where these assumptions were not satisfied, the Kruskal–Wallis test was applied. When significant differences were detected (*p* < 0.05), Fisher’s LSD test was used for pairwise comparisons. Analyses were performed in SPSS 22.0 (IBM Corp., Armonk, NY, USA), and results are presented as mean ± SE. The relationships among the physicochemical parameters were evaluated using Pearson’s correlation coefficient (r). Correlation coefficients were interpreted as follows: 0.00–0.29—weak correlation; 0.30–0.49—moderate correlation; and 0.50–1.00—strong correlation. Positive values indicate a direct relationship, while negative values indicate an inverse relationship. The magnitude of the correlation coefficient reflects the strength of the association [40].

## 3. Results

### 3.1. pH, Acidity (% Lactic Acid), Dry Matter, and Ash Contents

The effects of boric acid, salt, and their combination on the chemical properties of sheep milk are summarized in Table 1. Significant differences were observed in the milk’s pH between the treatment groups (*p* = 0.006). The C group exhibited the highest pH (6.88 ± 0.03), while the lowest pH was observed in the B40 group (6.62 ± 0.02). Groups S, B40, and BS20 had significantly lower pH values than C, whereas B20 and BS40 showed intermediate values. No significant differences were detected among the groups for acidity (% lactic acid) (*p* = 0.401), dry matter (*p* = 0.847), or ash content (*p* = 0.505). Acidity (lactic acid%) ranged from 0.21% to 0.36%, dry matter from 18.68% to 20.04%, and ash content from 0.76% to 0.99%, with minor variations between the treatments.

**Figure 1:**
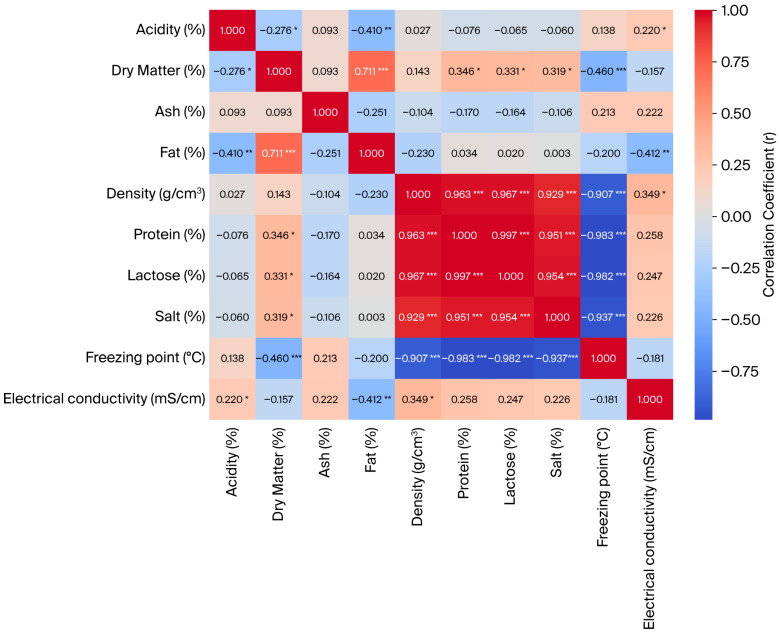
Correlation heat map illustrating the relationships among physical properties of sheep milk across experimental groups. Color intensity represents the strength and direction of Pearson correlation coefficients. (*: *p* < 0.05, **: *p* < 0.01, ***: *p* < 0.001).

Our analysis of the physicochemical properties of ewe milk revealed multiple significant correlations among the measured parameters (Figure 1). The pH showed negative correlations with dry matter (r = −0.332; *p* < 0.05) and electrical conductivity (r = −0.274; *p* < 0.05), indicating that higher acidity is associated with slightly lower pH and increased ionic content. Dry matter was strongly positively correlated with fat (r = 0.711; *p* < 0.01) and moderately correlated with protein (r = 0.346; *p* < 0.05), lactose (r = 0.331; *p* < 0.05), and density (r = 0.319; *p* < 0.05), while it showed a strong negative correlation with the freezing point (r = −0.460; *p* < 0.001), reflecting the effect of dry matter on lowering the freezing temperature. The ash content did not show significant correlations with other parameters (all *p* > 0.05), suggesting that the mineral content varied independently of major milk components. Fat was negatively correlated with the freezing point (r = −0.412; *p* < 0.01), indicating that higher fat content tends to lower the freezing temperature of milk. Milk density was very strongly correlated with protein (r = 0.963; *p* < 0.001) and lactose (r = 0.967; *p* < 0.001), and protein and lactose were highly correlated with each other (r = 0.997; *p* < 0.001), highlighting the interdependence of major milk solids on milk density. The salt content showed strong negative correlations with density (r = −0.937; *p* < 0.001) and protein (r = −0.983; *p* < 0.001), while its correlation with the freezing point was not significant (r = −0.181; *p* > 0.05). Electrical conductivity was negatively correlated with pH (r = −0.274; *p* < 0.05) and showed weak non-significant correlations with other parameters.

### 3.2. Results of Coagulation Time

The effects of boric acid and salt treatments on the coagulation time of sheep milk are presented in Table 2. The coagulation time varied among treatments, with the highest mean being observed in the BS20 group (995.03 s) and the lowest in the BS40 group (141.73 s). Coagulation did not occur in some groups, as indicated by their N/A values. The minimum and maximum coagulation times also showed considerable variation, ranging from 355 s to 2215 s among the treatment groups. However, the differences between the treatment groups were not statistically significant (*p* = 0.453, Kruskal-Wallis test), indicating that the treatments did not significantly affect the coagulation time under the conditions of this study.

### 3.3. Lactoscan Analyses of Milk

The effects of boric acid and salt addition on the chemical composition and physical properties of milk are presented in Table 3. Significant differences were detected in milk density (*p* = 0.048), protein content (*p* = 0.045), and salt content (*p* = 0.027). Specifically, the B40 group showed the highest protein content (4.98 ± 0.17%) and density (1.043 ± 0.046 g/cm^3^), while B20 had the lowest protein content (4.41 ± 0.12%) and density (1.037 ± 0.024 g/cm^3^). The salt content was significantly lower in the B20 group (0.94 ± 0.02%) compared with the C (1.06 ± 0.02%) and B40 (1.06 ± 0.03%) groups. No significant differences were observed among groups for fat (*p* = 0.712), SNF (solids-not-fat, *p* = 0.058), lactose (*p* = 0.059), freezing point (*p* = 0.102), or electrical conductivity (*p* = 0.634).

### 3.4. Results of Mineral Substance Analyses

According to the findings of this study, no statistically significant differences were observed between the treatment groups in terms of boron (B), calcium (Ca), copper (Cu), iron (Fe), magnesium (Mg), manganese (Mn), molybdenum (Mo), or zinc (Zn) levels (*p* > 0.05). However, a significant difference was found between the treatment groups in terms of selenium (Se) levels (*p* < 0.05). Specifically, selenium levels were higher in the B20 and B40 groups than in the control and salt groups, while lower values were found in the BS20 group (Table 4).

### 3.5. Results of Determination of Casein Fractions by SDS-PAGE Method

The alpha-, beta-, and total casein values of the treatment groups are presented in Table 5. Boron addition significantly affected the alpha-, beta-, and total casein levels in sheep milk (*p* < 0.001), and when evaluated in terms of OD%, the highest casein levels were observed in the control group and group B20, which were significantly higher than those of the other groups. Group S also maintained a relatively high casein level, although still lower than the C and B20 groups. In contrast, groups BS40 and BS20 showed significantly reduced casein values, while group C exhibited the lowest casein level of all the treatment groups. SDS-PAGE patterns of sheep milk proteins of the treatment groups are presented in Figure S1 as Supplementary Materials.

## 4. Discussion

### 4.1. pH, Acidity (Lactic Acid%), Dry Matter, Ash, and Fat Content

Milk’s pH is a critical parameter for lactation physiology, as well as the stability, enzymatic activity, and coagulation properties of milk proteins. Even small changes in pH can have significant consequences on product quality by affecting the surface charge of casein micelles, ionic balance, and milk buffering capacity [41]. In this study, changes in milk pH following the combined or separate application of boric acid and salt can be considered an indicator of ionic interactions that affect the biochemical structure of the milk. The effect of boron on milk composition is mainly related to mechanisms such as ion transport, enzyme activation, and membrane permeability at the cellular level [42]. The capacity of boron to form complexes with hydroxyl ions may enhance the susceptibility of milk proteins to fluctuations in environmental pH. This may play a role in the colloidal stability and calcium phosphate balance, particularly of caseins [43]. The effects of sodium chloride addition on milk’s composition are mainly explained through ionic strength and osmotic pressure. Salt can affect ion transport mechanisms by changing the sodium–potassium balance in epithelial cells that are involved in milk synthesis. This may reduce the buffering capacity of milk, leading to a decline in pH. In addition, the effect of salt on the solubility of calcium and magnesium ions may regulate the participation of milk proteins in caseinate formation and mineral-protein interactions. The dry matter and ash contents of milk are closely related to the nutritional status, water balance, and mineral metabolism of the animal [44]. Although boron and salt additives do not directly affect energy and protein metabolism, they may have indirect effects through the mineral cofactors of enzyme systems that are involved in milk synthesis. In particular, boron supports the activity of ATPase enzymes by increasing the bioavailability of magnesium, thus providing energy support for milk synthesis. The observed physicochemical effects of boric acid and salt on sheep milk play a regulatory role in its ionic balance and protein stability. It is thought that these interactions may have indirect effects on the processing properties of dairy products, especially their coagulation time, cheese yield, and textural quality. Therefore, the use of boron and salt at controlled levels may provide a technological advantage, both in terms of preserving milk composition and improving product quality. Recent studies on sheep milk’s composition indicate variability in pH, dry matter, and ash content depending on breed and production conditions. Çelik et al. [45] reported the pH value in sheep milk to be 6.55. Dagdelen and Esenbuga [46] reported ash values within the typical range for Morkaraman and Tushin sheep, highlighting breed-related differences in mineral content. Koca et al. [47] provided updated pH measurements for Norduz sheep milk, noting values that were consistent with the expected acidity profile of healthy and hygienic milk. Similarly, Turgut et al. [48] observed comparable pH levels in Hamdani crossbred sheep, further supporting the notion that the pH remains relatively stable across breeds under standard management conditions. Together, these recent findings demonstrate that while the pH tends to show limited variation among breeds, dry matter and ash values may differ more noticeably, reflecting genetic and environmental influences on milk composition. These results indicate that boric acid and salt treatments influenced the milk’s pH but did not significantly alter other chemical composition parameters under the conditions of this study.

In this study, the relationships between various physicochemical properties of sheep milk were examined, and the obtained correlation coefficients revealed important findings about the interactions between milk components. The results reveal highly significant and strong positive correlations, especially between protein, lactose, salt, and density. This suggests strong interrelationships among milk components and supports the use of density as a general marker of milk composition. The strong correlations between density and protein, lactose, and salt indicate that the density increases with increasing milk solid contents. Similarly, studies by Alichanidis and Polychroniadou [49] and Park et al. [50] reported that milk density is highly correlated with dry matter and protein contents. Negative correlations between the freezing point and milk components (especially with protein and lactose, respectively) are noteworthy. This finding indicates that the freezing point shifts to lower values as the milk solids content increases and suggests that freezing point measurement may serve as an indirect indicator for determining milk’s composition. This result has also been reported in similar studies on cow’s milk [14,51].

Relationships between fat percentage and other parameters were generally weak, and in some cases, they were negative. This demonstrates that the fat phase lowers both the density and electrical conductivity of milk. Furthermore, the strong positive correlation between dry matter and fat indicates that the increase in milk solids is largely dependent on the fat content. These findings are consistent with studies reporting strong positive correlations between fat content and dry matter in sheep milk [7,52].

Correlations between pH and other components were generally found to be low. However, the significant negative relationships between pH and ash and electrical conductivity indicate interactions between milk’s ionic composition and acidity. This result is consistent with studies indicating that ion mobility and, hence, conductivity decrease with increasing milk acidity [53,54].

Overall, these results indicate that dry matter, protein, and lactose are key contributors to milk’s density, while fat and dry matter influence its freezing point. The correlations also suggest moderate associations between pH and electrical conductivity, reflecting the ionic composition of the milk. These findings provide insight into the complex physicochemical relationships within ewe milk.

In general, the obtained correlations show that there are quite strong relationships between the various components of sheep milk, and especially parameters such as density, freezing point, and electrical conductivity can be considered indirect indicators of milk’s quality. These findings emphasize the importance of evaluating physicochemical parameters together in milk processing and quality control processes. The correlation analysis can be a useful tool for understanding changes in milk components and optimizing quality.

These results indicate that boric acid and salt treatments influenced certain physical and chemical parameters of milk, particularly protein, density, and salt content, while other parameters remained largely unaffected under the conditions of this study.

### 4.2. Coagulation Time

In several samples, particularly in those treated with boric acid alone, coagulation did not occur within the observed time period. This finding suggests that boric acid may interfere with the milk’s ability to form a proper gel matrix, possibly due to its interaction with casein micelles or calcium ions. Similar inhibitory effects of boron compounds on protein aggregation have been described in other food matrices [55]. The absence of coagulation in some boric acid groups indicates a potential destabilization of the micellar structure, reducing the efficiency of rennet activity [56]. Çelik and Özdemir [57] reported coagulation times within a typical range for sheep milk. In a subsequent study [58], found that coagulation characteristics varied between the Awassi and Morkaraman breeds and were influenced by different β-lactoglobulin variants. These studies collectively indicate that both breed and genetic polymorphisms can affect milk’s coagulation properties. The lack of coagulation observed in the B40 group may be related to boron-induced changes in mineral balance and casein micelle stability, which are critical for calcium-dependent coagulation. However, the underlying mechanism could not be fully clarified in the present study.

### 4.3. Lactoscan Contents of Milk

The modest fluctuations in protein and density values between treatments suggest a dose-dependent response. At low concentrations, boric acid may destabilize protein micelles, whereas at higher doses, especially in combination with salt, it could partially restore balance by enhancing ionic strength. This dynamic has been observed in mineral fortification studies, where the equilibrium between calcium, sodium, and phosphate strongly influences the structure and viscosity of milk. The current results are consistent with reports indicating that even small ionic perturbations can alter milk’s physical stability and gel-forming ability [56,59]. Recent studies investigating the composition of sheep milk show notable variation across breeds and production conditions. Çelik et al. [45] reported fat, SNF, protein, lactose, and freezing point values of 7.93%, 11.88%, 4.96%, 5.99%, and −0.615 °C, respectively. Koca et al. [47] reported that Norduz sheep milk contained relatively little fat (2.48%) but had a high SNF content (10.76%), with a specific gravity of 1.039 and protein and lactose levels of 5.09% and 4.79%, respectively. The freezing point was recorded as −0.602 °C, and the conductivity was 4.41 mS/cm. Similarly, Turgut et al. [48] observed that Hamdani crossbred sheep milk had a higher fat content (7.49%) and lower SNF content (8.69%), along with a density of 1.027, protein content of 4.13%, lactose content of 3.89%, and freezing point of −0.50 °C. The most recent data presented by Mutlu et al. [60] for Akkaraman sheep showed 5.62% fat and 12.01% SNF, with protein values reaching 5.69%. These recent findings collectively demonstrate considerable variability in fat, SNF, protein, lactose, and physical characteristics of sheep milk depending on the breed and study conditions, underscoring the importance of considering genetic and environmental factors when comparing the results of the present study.

### 4.4. Mineral Substance Content of Milk

Milk is an essential biological fluid for transporting several macro- and microminerals that are necessary for offspring development and maintenance of metabolic functions. Therefore, a change in milk’s mineral composition is an important indicator for understanding both the mother’s mineral metabolism and the physiological effects of dietary additives [61]. The milk minerals evaluated in this study reveal the interactive effects of boron and salt additives in particular. Boron is a microelement that can play a regulatory role in various mineral metabolisms, even at very low concentrations. It is known to be particularly effective on the homeostatic balance of calcium, magnesium, and phosphorus and plays a role in bone mineralization and cellular signal transmission [43]. Although the findings were not statistically significant, the increasing trend in Ca and Mg levels in the boron-added groups may be related to boron’s facilitative effects on calcium transport and the regulation of bone-derived mobilization. This may be explained by boron’s regulatory role in parathyroid hormone secretion and vitamin D metabolism. The observed variations in Cu, Fe, and Zn levels indicate that boron may indirectly influence metal-binding proteins and metallothionein activity. There is evidence that boron alters the intracellular transport of heavy metal ions by affecting metallothionein gene expression [62]. This mechanism may explain the observed intragroup differences, especially in Zn levels. Likewise, the decreased Cu and Fe levels in groups that were added with both boron and sodium chloride may result from changes in their intestinal absorption, mediated by competing carrier proteins, such as Divalent Metal Transporter 1 (DMT1). The most notable results were observed in selenium (Se) levels. Selenium plays a key role in regulating oxidative balance as a component of antioxidant enzymes, such as glutathione peroxidase (GSH-Px). The increase in milk Se levels in the groups receiving boron alone may indicate a positive effect of boron on redox systems. It has been previously stated that boron supports catalase and GSH-Px activities, which play a role in hydrogen peroxide destruction, thus increasing the bioavailability of selenium. Nonetheless, the reduction in Se levels in groups that were added with both boron and salt indicates a possible antagonistic effect, as high sodium chloride intake can impair selenium absorption or increase its renal elimination [23]. Thus, the beneficial effects of boron may be attenuated when co-administered with salt. Although boron and salt-individually or combined-exert only minor effects on milk’s overall mineral profiles, the notable alterations in Se metabolism indicate that these elements could modulate milk’s composition via oxidative stress and antioxidant defense pathways. These findings suggest that boron may act as a regulatory micronutrient for mineral balance and redox homeostasis, particularly during lactation. Previous studies reporting mineral compositions of sheep milk show considerable variation depending on breed and production conditions. Çelik and Özdemir [57] reported that Morkaraman sheep milk contained moderate levels of calcium (169.17 mg/kg), along with measurable concentrations of magnesium (17.78 mg/kg), sodium (77.74 mg/kg), potassium (91.31 mg/kg), and phosphorus (121.45 mg/kg), while other minerals were not measured. In a more recent study, Özkaya et al. [63] found substantially higher mineral concentrations in Akkaraman sheep milk, particularly for calcium (2077.37 mg/kg), magnesium (187.75 mg/kg), and phosphorus (1539.75 mg/kg), and also reported zinc contents of 5.57 mg/kg. Mutlu et al. [60] reported copper levels of 0.248 mg/kg in Akkaraman sheep milk, although data on other minerals were not available. These findings collectively indicate that mineral content can vary widely across breeds and studies, which should be considered when comparing our results with the existing literature.

### 4.5. Casein Content of Milk

The highest casein values were observed in the two boric acid groups (20 mg/day and 40 mg/day), indicating that boron addition may have a stimulatory effect on milk casein synthesis. This finding is consistent with previous research reporting that boron can influence mineral metabolism, particularly calcium and magnesium homeostasis, both of which are essential for casein micelle formation and stability [62,64]. In animal models, boron addition has been shown to reduce urinary excretion of calcium and magnesium, enhance mineral retention, and improve phosphorus utilization, thereby supporting protein-mineral interactions [65].

The boric acid (40 mg/day) group also exhibited relatively high casein levels, suggesting that the beneficial effect of boron may be dose-dependent. This observation is in line with earlier studies, where boron intake improved mineral utilization and bone composition, whereas higher doses failed to provide additional advantages or even disrupted mineral balance [65].

The lack of blood biochemical or hematological measurements is a limitation of this study, as dietary salt and boron additions may affect systemic physiological responses and animal health. Although the applied doses were based on levels previously reported as safe in ruminants, the absence of blood parameters prevents a comprehensive evaluation of the physiological safety of these dietary additions. Future studies should include blood-based indicators to better assess animal health and welfare.

In addition, the lowest casein level was detected in the control group, while the boron plus salt groups also showed significantly reduced casein contents compared with the salt and boric acid addition (20 mg/day) groups. A high level of dietary salt has been reported to alter osmotic balance and impair mineral absorption, especially of calcium and magnesium, which are key cofactors in casein synthesis and micelle stability [64]. Furthermore, salt may influence hormonal pathways regulating protein metabolism. Previous studies in dairy systems have shown that salt can directly affect the hydration, solubility, and network structure of casein, thereby modifying the quality of dairy products [66]. Similarly, in vivo findings indicate that excess salt addition in dairy camels negatively impacts the composition and quality of their milk [44]. These reports support our results that salt in combination with boron can counteract boron’s positive effects on casein synthesis.

The current findings suggest that boron and salt addition individually promote casein synthesis in sheep milk, likely via improved mineral homeostasis and protein biosynthesis pathways, while their combination diminishes the casein content and reduces boron’s beneficial effects.

Milk yield is known to influence milk composition through dilution and concentration effects. The absence of individual milk yield data in the present study is a limitation, as it prevented correlation analyses between milk production and milk compositional parameters. Therefore, the observed effects of dietary boron and salt addition on milk composition should be interpreted cautiously. Future studies should integrate milk yield measurements to clarify production-composition relationships better.

## 5. Conclusions

This study demonstrated that the addition of boric acid and salt to the diet of sheep did not significantly alter the gross chemical composition of milk; however, it induced measurable changes in key physicochemical properties related to milk processing. Variations observed in pH, density, protein-related parameters, and rennet coagulation behavior indicate that mineral and ionic modifications can influence the technological quality of sheep milk, even in the absence of major compositional changes. In particular, higher boron inclusion levels were associated with altered coagulation properties, suggesting a potential interaction between boron and calcium dynamics in milk. These findings directly address the study objectives and highlight the importance of considering mineral additions when aiming to modulate milk processing characteristics.

## Figures and Tables

**Table 1:** Effects of boric acid and salt treatments on pH, lactic acid, dry matter, and ash content of sheep milk.

Treatment Groups	pH	Acidity (% Lactic Acid)	Dry Matter (%)	Ash (%)
C	6.88 ± 0.03 ^a^	0.21 ± 0.01	19.65 ± 1.26	0.76 ± 0.12
S	6.65 ± 0.05 ^b^	0.22 ± 0.01	19.82 ± 0.35	0.83 ± 0.11
B20	6.74 ± 0.07 ^ab^	0.36 ± 0.13	19.28 ± 0.65	0.89 ± 0.05
B40	6.62 ± 0.02 ^b^	0.22 ± 0.01	20.04 ± 0.73	0.90 ± 0.09
BS20	6.66 ± 0.03 ^b^	0.23 ± 0.01	18.68 ± 0.69	0.99 ± 0.05
BS40	6.72 ± 0.04 ^ab^	0.28 ± 0.13	19.78 ± 0.67	0.76 ± 0.10
*p*-value	0.006	0.401	0.847	0.505

^a,b^: Different superscripts in the same column indicate differences between the treatment groups. Data are presented as mean ± standard error. C: control (no additives); S: 10 g/day rock salt; B20: 20 mg boric acid/day; B40: 40 mg boric acid/day; BS20: 20 mg boric acid + 10 g rock salt/day; BS40: 40 mg boric acid + 10 g rock salt/day.

**Table 2:** Effects of boric acid and salt treatments on coagulation time of sheep milk.

Coagulation Time (s)	C	S	B20	B40	BS20	BS40	*p*-Value
Min	*	355.00	*	*	*	*	0.453
Max	875.00	1045.00	2110	*	2215.00	425.00	
Mean	291.73	703.33	813.36	*	995.03	141.73	

*: Coagulation did not occur; C: control (no additives); S: 10 g/day rock salt; B20: 20 mg boric acid/day; B40: 40 mg boric acid/day; BS20: 20 mg boric acid + 10 g rock salt/day; BS40: 40 mg boric acid + 10 g rock salt/day.

**Table 3:** Effects of boric acid and salt addition on chemical composition and physical properties of milk.

Treatment Groups	Fat (%)	SNF (%)	Density (g/cm^3^)	Protein (%)	Lactose (%)	Salt (%)	Freezing Point (°C)	Electrical Conductivity (mS/cm)
C	9.40	13.47	1.042 ^ab^	4.86 ^ab^	7.34	1.06 ^ab^	−1.02	4.32
S	8.90	13.32	1.042 ^ab^	4.84 ^ab^	7.25	1.05 ^ab^	−1.00	4.22
B20	9.27	12.14	1.037 ^b^	4.41 ^b^	6.63	0.94 ^b^	−0.91	4.47
B40	8.45	13.70	1.043 ^a^	4.98 ^a^	7.46	1.06 ^a^	−1.03	4.50
BS20	8.81	12.87	1.040 ^ab^	4.67 ^ab^	7.01	1.01 ^ab^	−0.96	4.57
BS40	9.38	13.37	1.041 ^ab^	4.86 ^ab^	7.32	1.04 ^ab^	−1.01	4.37
SEM	0.47	0.37	0.034	0.13	0.19	0.02	0.03	0.15
*p*-value	0.712	0.058	0.048	0.045	0.059	0.027	0.102	0.634

Values are presented as means. SEM: pooled standard error of the mean. Different superscript letters within the same column indicate significant differences among treatment groups (*p* < 0.05). SNF: solids-not-fat; C: control (no additives); S: 10 g/day rock salt; B20: 20 mg boric acid/day; B40: 40 mg boric acid/day; BS20: 20 mg boric acid + 10 g rock salt/day; BS40: 40 mg boric acid + 10 g rock salt/day.

**Table 4:** Mineral composition of sheep milk.

Minerals	C	S	B20	B40	BS20	BS40	SEM	*p*-Value
Boron (×10^3^ ppb)	1.88	1.82	1.98	1.95	1.94	1.99	0.03	0.178
Calcium (×10^6^ ppb)	0.80	0.78	0.83	0.84	0.80	0.90	0.02	0.227
Copper (×10 ppb)	5.15	5.26	5.41	4.67	3.67	3.42	0.07	0.690
Iron (×10^3^ ppb)	0.81	1.19	1.05	0.88	1.09	0.88	0.06	0.478
Magnesium (×10^5^ ppb)	1.18	1.09	1.15	1.19	1.03	1.32	0.04	0.151
Manganese (×10 ppb)	7.54	8.84	9.39	7.54	7.94	7.48	0.28	0.229
Molybdenum (×10 ppb)	4.72	4.46	4.22	4.62	3.98	4.80	0.03	0.594
Selenium (×10 ppb)	8.26 ^ab^	8.26 ^ab^	9.48 ^a^	9.55 ^a^	6.51 ^b^	7.78 ^ab^	0.03	0.026
Zinc (×10^3^ ppb)	4.43	5.33	5.04	4.93	3.95	5.28	0.17	0.147

Values are presented as means. SEM: pooled standard error of the mean. Different superscript let-ters within the same column indicate significant differences among treatment groups (*p* < 0.05). Mineral concentrations were scaled for clarity. Values are presented according to the multiplication factor indicated in the row headers. C: control (no additives); S: 10 g/day rock salt; B20: 20 mg boric acid/day; B40: 40 mg boric acid/day; BS20: 20 mg boric acid + 10 g rock salt/day; BS40: 40 mg boric acid + 10 g rock salt/day.

**Table 5:** Total casein contents in sheep milk from the treatment groups.

Treatment Groups	Alpha-Casein (OD%)	Beta-Casein (OD%)	Total Casein (OD%)
C	9.93 ^d^	4.97 ^d^	16.55 ^d^
S	14.57 ^b^	7.28 ^b^	24.28 ^b^
B20	15.60 ^a^	7.80 ^a^	26.00 ^a^
B40	16.17 ^a^	8.09 ^a^	26.95 ^a^
BS20	11.31 ^c^	5.66 ^c^	18.85 ^c^
BS40	12.16 ^c^	6.08 ^c^	20.26 ^c^
SEM	0.23	0.11	0.38
*p*-value	<0.001	<0.001	<0.001

OD%: optical density; Different superscript letters within the same column indicate significant differences among treatment groups according to Fisher’s least significant difference (LSD) test (*p* < 0.05). F value obtained from one-way ANOVA was 113.508; C: control (no additives); S: 10 g/day rock salt; B20: 20 mg boric acid/day; B40: 40 mg boric acid/day; BS20: 20 mg boric acid + 10 g rock salt/day; BS40: 40 mg boric acid + 10 g rock salt/day.

## Data Availability

The data presented in this research are available on request from the corresponding author.

## Supplementary Materials

The following supporting information can be downloaded at https://www.mdpi.com/article/10.3390/ani16020233/s1, Figure S1: SDS-PAGE patterns of sheep milk proteins of the treatment groups.

## Abbreviations

The following abbreviations are used in this manuscript:

TSI	Turkish Standard Institute
TS	Turkish Standard
AOAC	Association of Official Analytical Chemists
SNF	Solids-not-fat
SDS-PAGE	Sodium dodecyl sulfate polyacrylamide gel electrophoresis
ANOVA	Analysis of variance
SPSS	Statistical Package for the Social Sciences
TÜBİTAK	The Scientific and Technological Research Council of Türkiye
DMT1	Divalent Metal Transporter 1
ISO	The International Organization for Standardization
IDF	International Dairy Federation
