# Supplementary material for: Effects of Dietary Salt and Boric Acid on Milk Quality in Savak Akkaraman Sheep
Source: Animals (Basel). 2026 Jan 13;16(2):233. doi: 10.3390/ani16020233 (PMC12837315; doi:10.3390/ani16020233)
Supplement: Supplementary file 1 [file animals-16-00233-s001.zip › animals-4038932-supplementary.pdf]

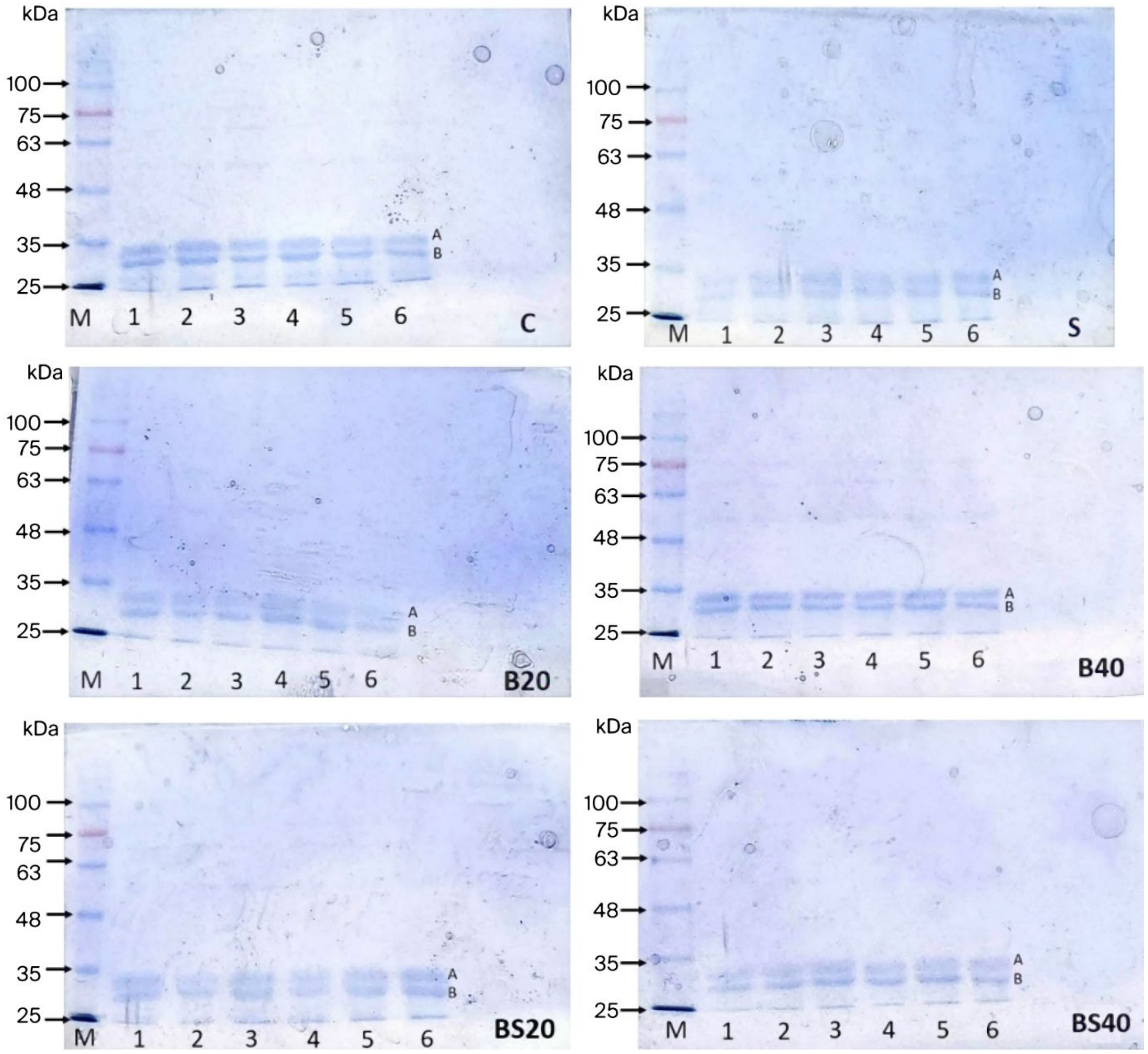

Figure S1. SDS-PAGE patterns of sheep milk proteins of the treatment groups. The lanes 1-6 represent individual samples. Major protein bands are observed mainly in the 25-35 kDa region. **M:** marker, **A:** alpha-casein, **B:** beta-casein; **C:** control (no additives); **S:** 10 g/day rock salt; **B20:** 20 mg boric acid/day; **B40:** 40 mg boric acid/day; **BS20:** 20 mg boric acid + 10 g rock salt/day; **BS40**: 40 mg boric acid + 10 g rock salt/day.
